# EGFR inhibitors: clinical aspects, risk factors and biomarkers for acneiform eruptions and other mucosal and cutaneous adverse effects^☆^

**DOI:** 10.1016/j.abd.2022.10.004

**Published:** 2023-03-27

**Authors:** Júlia Kanaan Recuero, Joana Roberta Fitz, Andrea Abe Pereira, Renan Rangel Bonamigo

**Affiliations:** a Postgraduate program in Pathology, Universidade Federal de Ciências da Saúde de Porto Alegre, Porto Alegre, RS, Brazil; b Dermatology Service, Irmandade Santa Casa de Misericórdia de Porto Alegre, Porto Alegre, RS, Brazil; c Universidade Federal do Rio Grande do Sul, Porto Alegre, RS, Brazil

**Keywords:** Acneiform eruptions, Drug-related side effects and adverse reactions, Risk factors, Skin manifestations, Target therapy

## Abstract

The frequency of the use of drugs that act on the epidermal growth factor receptor (EGFR) is increasing, with the consequent onset of cutaneous toxicity, specifically acneiform eruption. The authors extensively review the topic, focusing on describing how these drugs can affect the skin and its appendages, that is, the pathophysiology that encompasses the cutaneous toxicity related to the use of EGFR inhibitors. In addition, it was possible to list the risk factors that may be associated with adverse effects of these drugs. Based on this recent knowledge, the authors expect to aid in the management of patients who are more vulnerable to toxicity, reduce morbidities, and improve the quality of life of patients undergoing treatment with EGFR inhibitors. Other issues related to the toxicity of EGFR inhibitors, such as the clinical aspects of the acneiform eruption grades, and other different types of cutaneous and mucosal reactions, are also included in the article.

## Introduction

The skin is a relatively common target for adverse drug reactions, but it is difficult to estimate the exact incidence of these reactions. In hospitalized patients, data from the literature report that skin reactions are responsible for approximately 3% of disabling complications in inpatients, representing 2% of medical consultations and 5% of admissions to dermatology services^1^, ^2^.

Specifically, in oncology therapy, the number of newly available drugs is growing rapidly. Therefore, there is a constant change in the management of possible adverse effects. Several new drugs have been studied, and immunotherapy and targeted therapies have become the treatments of choice for many types of cancer^3^. Concomitantly with the increase in the use of these new treatments, the description of previously unknown or unusual skin reactions, which are different from those experienced with conventional chemotherapy, has been observed^3^.

Among these modern drugs, particularly targeted therapies, drugs directed to the epidermal growth factor receptor (EGFR) stand out. This receptor is expressed in many solid tumors and is also identified in keratinocytes present in the cutaneous epidermis, skin appendages, and the endothelium of dermal capillaries^3^. Therefore, EGFR inhibition therapy interferes with the signaling pathways in the epidermis and skin appendages. As a consequence, it becomes logical that one of the main toxicities of these inhibitors is concentrated in the cutaneous tegument.

Currently, epidermal growth factor receptor inhibitors (EGFRi) are used in the treatment of many malignant neoplasms located in head and neck, lung, large bowel, prostate, breast, ovary, stomach, and pancreas^4^. The EGFRi can comprise monoclonal antibodies against EGFR (cetuximab and panitumumab), EGFR-specific small molecule tyrosine kinase inhibitors (erlotinib and gefitinib), dual EGFR and HER2 kinase inhibitors (lapatinib, neratinib and afatinib), erbB receptor inhibitors (canertinib) and other less specific multikinase inhibitors (vandetanib).

Since these therapeutic agents are widely used and increasingly prescribed, it is important to acquire knowledge about the physiopathological mechanisms of skin, hair, nail and mucosal toxicities, as well as the risk factors for the development of reactions. By recognizing individual clinical susceptibilities, there is the possibility of anticipating the optimization of therapeutic management.

In patients undergoing treatment with EGFR inhibitors (EGFRi), the most common skin reaction is acneiform eruption. The present article shows and discusses the clinical aspects, and the main risk factors for this toxicity and briefly addresses other related skin contexts^5^.

## EGFRi and acneiform eruption

EGFR is expressed in a variety of normal skin tissues, including epidermal basal cells, sebaceous glands, outer root sheath cells, eccrine glands, and vascular smooth muscle cells^6^. Most EGFR-targeted agents produce a similar spectrum of dermatological toxicities^4^, ^7^.

EGFR stimulation regulates epidermal growth and inhibits keratinocyte apoptosis. EGFRs also affect the formation of sweat and sebaceous glands, as well as inhibit hair growth and participate in angiogenesis^4^. The inhibition of EGFR activity has a marked effect on epidermal homeostasis, resulting in pronounced thinning of the epidermis, including the stratum corneum. Moreover, it leads to a reduction in the protective function of the skin due to increased permeability, increasing the risk of bacterial infections. Cytokine release and the activation of cells involved in the inflammatory process are responsible for the susceptibility to skin toxicity when an EGFR blocker is used^4^, ^8^. The exact mechanism leading to skin toxicity during treatment with an EGFRi is not well known, but it is undoubtedly the result of genetic modifications of the signals associated with its activation. It is known to interfere with the RAS/RAF/MEK/ERK pathway, which affects cell cycle regulation, including epidermal cell proliferation and differentiation^4^.

The papulopustular rash is the most frequently reported skin toxicity following the use of an EGFRi. The overall incidence ranges from 60% to 80%^9^, reaching 90% of patients treated with cetuximab and panitumumab^10^.

The eruption can be monomorphic or pleomorphic and mainly affects seborrheic areas, such as the face, scalp, and upper thorax regions. In the literature, it is usually described as an acneiform eruption, but unlike acne vulgaris, comedones or purulent cysts are not found, so the specific treatment for acne vulgaris is not adequate^4^, ^8^, ^11^, ^12^.

In over 75% of patients, the initial skin lesions appear within the first two weeks of treatment. Erythema and edema usually appear first, accompanied by sensory disorders. Soon after, between the second and fourth week, folliculitis and/or pustular lesions with pruritus occur. Complete disappearance is observed approximately one to two months after drug withdrawal^4^, ^8^, ^11^, ^12^ and may cause post-inflammatory hyperpigmentation^4^, ^8^, ^11^. Pain and pruritus^13^ are common symptoms and crises may be induced by the medication with each administration^14^, ^15^.

### Classification with acneiform eruption grading

Cutaneous adverse reactions can be classified according to some scales. Among them, the NCI CTCAE system v5.0 (*Common Terminology Criteria for Adverse Events* version 5.0. [http://www.cancer.gov]), which classifies the eruption severity according to some variants: physical manifestation, psychosocial impact, effect on Activities of Daily Living (ADL) and need for intravenous antibiotic therapy. The calculation of the affected Body Surface Area (BSA), which uses the “Rule of 9’s”, can lead to confusion, as severe reactions that affect a small area of the body may be classified at a lower grade^4^, ^5^, ^16^, ^17^.

**Figure 1:**
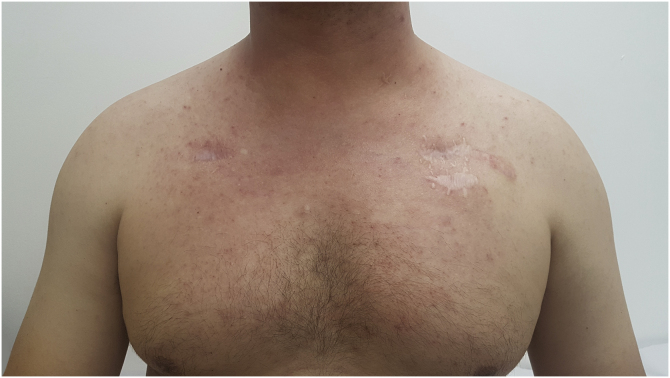
A 36-year-old patient using cetuximab with grade 1 acneiform reaction in CTCAE v5.0 grading criteria. Papules and pustules on the upper trunk, covering less than 10% of the body surface.

Grade 1 ‒ Papules and/or pustules covering <10% of the Body Surface Area (BSA), which may or may not be associated with symptoms of pruritus or tenderness (Fig. 1).

Grade 2 ‒ Papules and/or pustules covering 10%‒30% BSA, which may or may not be associated with symptoms of pruritus or tenderness; associated with psychosocial impact; limitation of instrumental Activities of Daily Living (IADL); papules and/or pustules covering > 30% of BSA with or without mild symptoms.

**Figure 2:**
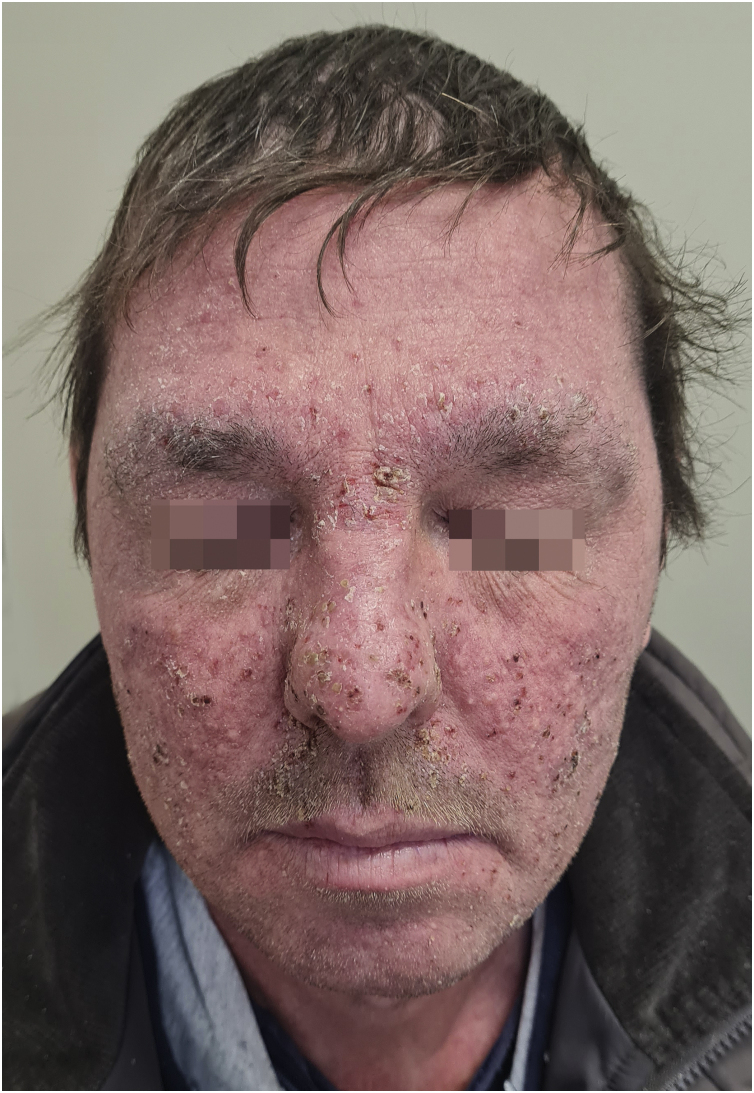
A 49-year-old patient using cetuximab with grade 3 acneiform eruption according to the CTCAE v5.0 grading criteria. Papules and pustules covering >30% of BSA, associated with moderate pruritus and pain, in addition to local superinfection.

Grade 3 ‒ Papules and/or pustules covering > 30% of the BSA, with moderate or severe symptoms; limiting ADL self-care; associated with local superinfection with the oral antibiotics used (Fig. 2).

Grade 4 ‒ Life-threatening consequences; papules and/or pustules covering any % of BSA, which may or may not be associated with symptoms of pruritus or tenderness and are associated with extensive superinfection with the intravenous antibiotics used.

Grade 5 - Death.

A second classification system is the MESTT (Multinational Association of Supportive Care in Cancer - MASCC; EGFR Inhibitor Skin Toxicity Tool), a complex system that takes into account data reported by the patient, such as changes in quality of life, changes in the time and dose of treatment as related to the adverse effect^16^.

There is also the three-part system proposed by Wollenberg et al. The Induced Rash Severity Score (IRSS or WoMoScore), a specific scoring system introduced in 2008^18^ that combines the severity of five EGFRi eruption characteristics (color of eruption, distribution of eruption, papulation, pustulation, and desquamation/crusts), associating the score based on the extent of the affected facial area and the total body area involved. The final score ranges from 0 (no skin disease), 1 to 20 (mild), between 20 and 40 (moderate), to scores above 40 points in severe cases^16^.

Severity is based on the BSA involvement and the degree of ADL limitation^7^. A severe rash occurs in 10% of patients. When severe, dermatologic toxicities can lead to dose modification or discontinuation in 36% and 72% respectively, by healthcare professionals. Although the side effect profile may be primarily dermatological, the toxicities result in significant physical and emotional discomfort; therefore, maximizing supportive measures is crucial^19^.

Histopathologically, the analyses performed in patients with acneiform eruptions have shown a superficial inflammatory infiltrate around the hyperkeratotic or ectatic follicular infundibulum or suppurative neutrophilic folliculitis with disruption of the epithelial lining^15^, ^19^. Basal keratinocytes and hair follicles show higher levels of p53^15^. The pustules are sterile, showing negative cultures for bacteria, fungi, yeasts, and absence of *Demodex folliculorum*^14^.

### Risk factors for acneiform eruption

The relevant risk factors for acneiform eruptions are shown in Table 1 and will be discussed separately below.

**Table 1:** Risk factors for acneiform eruptions associated with EGFRi use.

**Related to treatment**

Type of medication, dose and duration
Ionizing radiation
**Related to the tumor**
**Related to the patient**
Phototype
Age
Sex
Ethnicity
Sebaceous secretion
Presence of *Demodex*
Immune system
Bacterial infection
**Environmental factors**
UV radiation
Sensitizers
**CPK levels**
**Biomarkers and genetic polymorphisms**

### Type of medication, dose and duration of treatment

The occurrence of an eruption depends mainly on the type and dose of the medication used. The literature suggests that the frequency^19^ and severity of the eruption depend on the type of drug used^10^. Monoclonal antibodies result in reactions more frequently^4^, ^19^ than tyrosine kinase inhibitors, for instance^4^.

Owczarek et al. reported the occurrence of eruption in 88% to 90% of patients using cetuximab, 100% of patients using panitumumab, 43% to 54% of gefitinib users, 75% of erlotinib users, and 13% to 47% of lapatinib users^4^.

Some evidence indicates that the incidence and severity/intensity of the reaction is dose-dependent, as well as the duration of the cutaneous condition^9^, ^20^. According to Macdonald, increasing the dose and restarting the medication can increase the crises^7^.

### Ionizing radiation

The sensitivity of EGFRi-treated cells to ionizing radiation has been documented. Treatment with EGFRi before radiotherapy increases the sensitivity of tumor cells to radiation. Interestingly, areas of previously irradiated skin may not develop an eruption during EGFRi use. Chronic radiation-induced alterations, such as the absence of follicles and sebaceous glands, resulting from the induction of apoptosis and fibrosis by radiotherapy, explain this finding^21^.

Bossi et al. evaluated six patients with cetuximab-induced acneiform eruption and found that previously irradiated areas were free of skin lesions^22^.

Other investigators also claim that sites previously treated with radiation are typically spared during the occurrence of acneiform eruptions^7^, ^14^. Yalçin reported on a patient with non-small cell lung cancer (NSCLC) on erlotinib who developed a typical acneiform eruption that spared the area previously submitted to radiotherapy. A biopsy of the affected area and the spared area (previously irradiated) was performed. In the first, histopathology disclosed suppurative folliculitis destroying the follicular epithelium and adnexal structures. In the second, there was no follicular structure. The estimated dose for hair follicle involvement in radiotherapy is about 40 Gy (the patient received 45 Gy in total for the neoplasm, whereas the normal skin received 18.5 Gy)^23^. It is concluded that the patient did not have acneiform eruption in the area previously submitted to radiation therapy due to follicle loss, which is very similar to the pathogenesis of acneiform eruption in patients using EGFRi.

In contrast, Tejwani performed a meta-analysis to assess acneiform eruptions in 1,238 patients treated with EGFRi with or without radiotherapy^24^. The results showed a higher incidence of skin reactions in patients who received combined therapy (EGFRi and radiation). Regarding the medications, the highest incidence was observed in patients receiving therapy with erlotinib plus cisplatin, and the lowest incidence was in patients receiving cetuximab plus docetaxel/cisplatin.

Wu proposed that significant “field toxicity” can occur when EGFRi are used concomitantly with radiotherapy, as the radiation causes an upregulation of EGFR in normal skin^17^. A randomized phase 3 trial, which compared radiotherapy with or without cetuximab in patients with locoregionally advanced head and neck squamous cell carcinomas, showed an extension of the radiodermatitis duration in part of the study group that used cetuximab^15^.

### Cancer type

Su published a systematic review and meta-analysis that quantified the overall incidence and risk of severe cutaneous eruption in patients with different types of solid tumors treated with cetuximab. A total of 2,037 patients were analyzed and the overall incidence of acneiform eruptions was 81.6%, with 6.5% being high grade. Although not statistically significant, a high incidence of acneiform eruptions was observed in patients with colorectal cancer (CRC) compared to non-CRC (6.8 vs. 5.6%; RR = 1.2; p = 0.402). In other words, the study indicated that the risk of developing a high-grade rash varies according to the type of tumor. This is possible due to the diversity of neoplasms and their biological differences (such as growth factor secretion), which may affect skin susceptibility to toxicity^25^. Therefore, the rash grade may reflect interactions not only between the skin tissue and the drug but also between the skin tissue and the tumor^26^.

### Phototype

Several authors have reported that patients with a low phototype ‒ fair skin ‒ are more prone to developing EGFRi eruptions, in addition to having more intense reactions^10^, ^14^, ^27^. These data were described by Lacouture 2011 only with the use of erlotinib in low phototypes^19^. This is in contrast to other evidence, which found no association between patient phototype and reaction severity^20^, ^26^.

### Age and sex

Regarding age and sex, data suggest that younger and male patients are at greater risk of developing the eruption^27^, ^28^. It is also known that, with advancing age, cultured fibroblasts express fewer epidermal growth factor receptors and thus, EGFRi may have fewer cutaneous targets in older patients and therefore show less cutaneous toxicity^28^.

Jatoi investigated clinical predictors of severe cutaneous eruption in 933 patients treated with cetuximab as adjuvant chemotherapy for colon cancer. Fifty patients (5%) developed a severe rash, and more men than women developed this eruption: 34 (7%) vs. 16 (3%). A greater number of young patients (< 70 years of age) also developed an eruption: 48 (6%) vs. 2 (1%) who were over 70 years old and male^19^, ^28^. As supported by Le-Rademacher et al., the male sex is two-fold more likely to develop EGFRi-induced grade 3 or higher eruption than the female sex^29^.

Moreover, hormones constitute another factor that may be involved in the development of gender-related eruptions. Androgens and estrogens seem to interact with the epidermal growth factor receptor^28^, ^29^. One article described the importance of androgens and showed that the related androgen receptor genes are overexpressed in the skin of patients with an EGFRi reaction^29^.

Racial diversity was unrelated to the risk of skin eruption^9^, ^28^. Other studies have not found sufficient results to correlate age, sex, or other individual characteristics with susceptibility to skin reactions^9^.

It should be mentioned that dry skin, commonly seen in older patients, can lead to the development of an eruption. Considering this hypothesis, older patients with a history of atopic dermatitis and people who have undergone previous therapy with cytotoxic agents would have a higher theoretical risk of developing this eruption^30^.

### Sebaceous secretion

The literature findings are conflicting on this topic. For some authors, increased sebum production is not associated with an increased risk for skin toxicity. However, caution is necessary, as a prior predisposition to folliculitis and acne may be associated with cutaneous adverse events during the use of EGFR inhibitors^7^.

Nakahara conducted a study to determine the relationship between skin sebum levels and acneiform eruption by measuring sebum levels before and after treatment with EGFRi. Eight patients with non-small cell lung cancer (NSCLC) who received gefitinib or erlotinib were evaluated for sebum levels on the face, chest, and dorsal regions before and after therapy. Patients who already had elevated sebum levels or had a major change in relation to the pretreatment baseline developed acneiform skin eruptions. This result may reveal that sebaceous gland activity can be involved in the mechanism underlying the origins of acneiform eruptions (AfE) in patients treated with these drugs^6^.

Kikuchi et al. sought to elucidate useful and highly sensitive parameters for the early detection of skin changes caused by EGFRi. Transepidermal water loss, skin surface hydration, skin surface lipid levels, and erythema/melanin index were serially measured for two weeks in 19 patients treated with afatinib/erlotinib (EGFR-TKI) and for eight weeks in 20 patients treated with cetuximab. The levels of transepidermal water loss on the cheek in patients who developed grade 2 or higher acneiform eruptions were elevated within seven days of starting afatinib/erlotinib therapy compared with those before therapy, as well as in patients with grade 1 or lower. In patients treated with cetuximab, skin surface hydration on the cheek in patients with grade 2 or higher toxicity was significantly decreased when compared to patients with grade 1 reactions at two and six weeks. Baseline skin surface lipid levels and cheek erythema index of patients with AfE ≥ Gr2 were significantly higher than those with AfE ≤ Gr1. In conclusion, the instrumental assessment found rapid inflammatory skin alterations by EGFRi and identified oily skin as a risk for severe AfE^31^.

Further studies have been performed, and in one of them, in which ten patients with renal cell carcinoma were treated with cetuximab, no apparent association was found between reaction severity and skin type or history of acne^32^.

According to Sipples, when using TKI, the reaction is more intense in patients with oily and acne-prone skin^12^. Other authors have not associated skin type, history of acne, or rosacea with the onset and severity of EGFRi use reactions^20^.

### Demodex folliculorum

Regarding *Demodex folliculorum*, the evidence found is contradictory. Gerber analyzed through biopsy the density of *Demodex folliculorum* in lesions of 19 patients with EGFRi-induced eruption. The collected data have shown that patients on EGFRi therapy are more susceptible to mite colonization or infection^33^. However, other studies failed to detect the mite skin biopsies of ten patients with cetuximab-induced acneiform eruption and concluded that the eruption is not related to the presence of *Demodex*^34^.

### Patient immune system

One article observed that patients with better immune performance and better immune systems have more intense reactions^10^.

### Bacterial infection

Some authors have claimed that EGFRi not only induces inflammation but also suppresses the synthesis of antimicrobial peptides and skin barrier proteins in keratinocytes ‒ which predisposes to bacterial infections^35^. Interesting articles have identified bacterial infections at cutaneous toxicity sites in approximately one-third of cancer patients treated with EGFR inhibitors, and most of these patients also had a positive blood culture for *Staphylococcus aureus*^8^.

Tohyama et al. evaluated the incidence of bacterial infection and treatment outcomes in patients with advanced-stage papulopustular eruption. Bacterial culture was performed in 51 cases, 50 of which yielded positive results. After topical and/or oral antibiotic therapy without the use of topical corticosteroids, the papulopustular eruption improved rapidly. However, the use of a combination of topical antibiotics and corticosteroids prolonged the recovery period. In conclusion, folliculitis that develops more than four weeks after starting treatment with an EGFR inhibitor is typically caused by a staphylococcal infection. Bacterial culture is necessary due to the high levels of antibiotic resistance. It is important to differentiate between sterile pustules (more common in the early phase of the eruption) and infectious pustules (more common in the late phase), as the treatment of these conditions is different, and the incorrect use of topical corticosteroids can prolong or transform infectious recurrent pustular eruptions^35^.

It is already known that acneiform eruptions occur through the inhibition of EGFR in keratinocytes, but the overlap of a bacterial infection may play an important role^36^. Lacouture reported bacterial infection in skin lesions in one of three patients treated with EGFRi^8^, ^21^. *Staphylococcus aureus* was identified in most of them, which is something found in data on the prevalence of these microorganisms in EGFRi eruptions. Some authors indicate antibiotic prophylaxis as a protective factor against the generation of acneiform eruptions in patients treated with EGFRi^17^, ^37^.

### Ultraviolet radiation

EGFR inhibition enhances ultraviolet (UV) radiation-induced keratinocyte apoptosis. Under physiological conditions, UV radiation damages the DNA of keratinocytes, affecting the formation of free radicals. Consequently, there is an increase in EGFR expression and an increase in proliferative signals. Disturbances of this process can cause lesions or exacerbations induced by exposure to ultraviolet radiation. In hair follicles, this process results in increased expression of genes that stimulate inflammatory processes, apoptosis, and duct blockage, leading to rupture^4^, ^21^. Thus, UV radiation, combined with EGFR inhibition, can cause additional oxidative stress and inflammatory response in keratinocytes, contributing to the pathogenesis of the eruption.

A prevention study was conducted, in which 54 patients submitted to EGFR-TKIs or anti-EGFR monoclonal antibodies were tested for sunscreen use. The incidence of skin eruption at eight weeks was 78% and 80% with sunscreen and placebo, respectively (p = 1.00). Thus, sunscreen may be effective if combined with other methods^3^.

Although ultraviolet radiation can exacerbate skin reactions, studies have not shown that photoprotection prevents their development^4^. The use of sunscreen in a placebo-controlled study did not prevent or attenuate EGFR inhibitor-induced eruption^7^. Another four authors also pointed out sun exposure as a triggering and/or aggravating factor of the eruption^7^, ^10^, ^14^.

### Sensitizers

The skin of these patients is abnormally sensitive to irritants and allergens. EGFR inhibition can result in the erroneous proliferation, migration, and differentiation of target cells and lead to disruption of skin integrity, with the recruitment of inflammatory cells^38^.

Several skin care procedures are recommended, such as cleansing the skin with bath oil in cold or lukewarm water, using an alcohol-free emollient to retain moisture, and photoprotection. In case of exposure to sunlight, the use of sun protection products with a high protection factor is recommended. Treatment with corticosteroids is not recommended, as these medications can induce rosacea. Although effective for acneiform eruptions, benzoyl peroxide is often an irritant. Topical retinoids are not recommended as well, as the mechanisms of anti-EGFR and acne vulgaris eruption are different and they may also aggravate the skin irritation^11^.

Waris et al. reported a case of a 61-year-old African-American male with locally advanced oropharyngeal cancer who was treated with cetuximab and radiation. He developed a sudden eruption after the 5^th^ cycle of cetuximab while using over-the-counter skin care medication. Topical agents should be used with extreme caution in patients receiving anti-EGFR therapy. Over-the-counter topical acne and dry skin medications can suddenly change a mild form of acne into a severe cutaneous toxic skin eruption associated with intense desquamation and exfoliation^38^.

### Creatine kinase levels

Elevated serum creatine kinase levels have been associated with skin eruption severity and may have the potential to predict which patients will develop skin lesions^38^. Creatine (Cr) and creatine kinase (CK) play an important role in human soft tissues. At a cellular level, CK catalyzes the reversible phosphorylation between ATP and Cr to produce phosphocreatine (PCr) and ADP, acting like a cytosolic energy buffer. During wound healing, keratinocytes overexpress CK-BB to cope with the increased metabolism caused by nucleotide biosynthesis. It has been hypothesized that the cutaneous toxicity caused by new anticancer agents is associated with keratinocyte stress, leading to increased CK levels in the skin and possibly increased CK levels in circulating plasma. Garcia et al. investigated the association between skin eruptions and plasma creatine kinase (CK) levels and concluded that the increase in plasma CK is associated with the development of eruptions caused by EGFRi^39^.

### Biomarkers and genetic polymorphisms

Some biomarkers and genetic polymorphisms studied as possible risk markers for the development of acneiform eruptions when using EGFRI are depicted in Table 2 and discussed below.

**Table 2:** Biomarkers and mutations predictive of the severity of anti-EGFR-related toxicity.

Reference	Biomarker/Genetic polymorphism	Risk factor for toxicity
Vallböhmer et al. (2005)	Cyclooxygenase-2	Low expression
Tan et al. (2008)	pAkt	Low expression
Graziano et al. (2008)	EGFR intron 1 (CA) repeat	Low expression
Parmar et al. (2013)	PIK3CA	Mutation
Jaka et al. (2014)	SNP-216	Mutation
Paul et al. (2014)	CXCL8 (IL-8)	Low serum levels
Takahashi et al. (2014, 2015)	Epiregulin (EREG) Amphiregulin (AREG)	Low serum levels
	HGF	
Hichert et al. (2017)	HGF	High serum levels
Froelich et al. (2018)	SNP- rs849142	Mutation
Labadie et al. (2021)	RARA	Mutation

Kubo reviewed previous studies and found several associations, described in Table 2, together with other findings on biomarkers predictive of the severity of anti-EGFR toxicity in colorectal cancer^37^.

Hichert et al. are also mentioned, as their article showed that patients with elevated serum levels of hepatocyte growth factor (HGF) developed less severe acneiform eruptions^40^.

In the study by Froelich et al., the single nucleotide polymorphism (SNP) rs849142 was significantly associated with acneiform eruptions in patients treated with EGFR^41^.

Tan reported that patients with low phosphorylated RAC-α serine/threonine protein kinase (pAkt) activity are more likely to develop acneiform skin toxicity when using EGFRi. Adding a MAPK inhibitor to the therapy of these patients may help control the skin eruption^42^.

Paul et al. showed that low serum C-X-C motif ligand 8 (CXCL8) ‒ a potent angiogenic factor ‒ was associated with greater severity of acneiform eruptions^43^.

Moreover, Jaka et al. suggested that the SNP-216 polymorphism of the EGFR gene may be useful in anticipating treatment response and the onset of severe acneiform eruptions^44^. Two genetic variants of the retinoic acid receptor alpha (RARA) gene have been significantly associated with critical skin toxicity (including patients with desquamation and acneiform skin eruptions). This finding may represent a potential therapeutic target for prophylactic or reactive treatment in patients undergoing this therapy^45^.

Parmar et al. described more than one genetic polymorphism correlated with skin toxicities but did not specify the association with acneiform eruptions, only with general toxicity^46^.

### Treatment and prophylaxis

**Figure 3:**
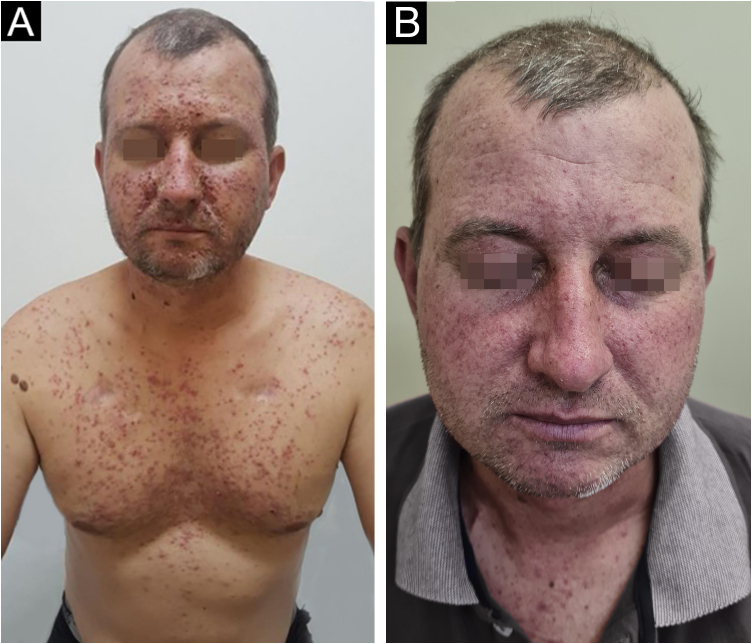
(A) A 40-year-old patient with grade 3 acneiform eruption due to the use of panitumumab for colorectal cancer. **(B)**The same patient after 14 days using doxycycline 100 mg twice daily.

A multinational, interdisciplinary group of experts in supportive care in cancer reviewed relevant studies using established criteria to develop recommendations for dermatologic toxicities associated with EGFR inhibitors. Based on the higher frequency of eruptions in patients treated with EGFR inhibitors and the consistent occurence within the first two to four weeks of therapy, prophylactic management is recommended, unless there are contraindications based on patient and/or healthcare professional factors. Hydrocortisone 1% combined with skin moisturizer, sunscreen, and doxycycline 100 mg twice daily is recommended for the first six weeks, based on randomized data. Another study found prophylactic minocycline 100 mg daily to be an effective agent in reducing the number of lesions during the first eight weeks. Doxycycline seems to have a more favorable safety profile, especially in patients with renal dysfunction (Fig. 3). Meanwhile, minocycline is less photosensitizing and therefore preferable in geographic locations or during seasonal with a high UV index^19^.

The reactive use of medium-to-high potency topical corticosteroids is recommended based on studies showing the in vitro release of inflammatory chemokines after therapy with EGFR inhibitors. Vitamin K3 (menadione) is being studied, but published reports of vitamin K1 are based on studies without control groups. Similarly, studies investigating isotretinoin for the treatment of eruptions induced by EGFR inhibitors did not include control groups, but consistent reports of isotretinoin use at doses lower than the ones used for acne support the recommendation for its use when other measures have failed^19^.

### Other reactions

In addition to acneiform eruptions, other skin toxicities can occur with the use of EGFRi; they are briefly discussed below.

### Xerosis and pruritus

**Figure 4:**
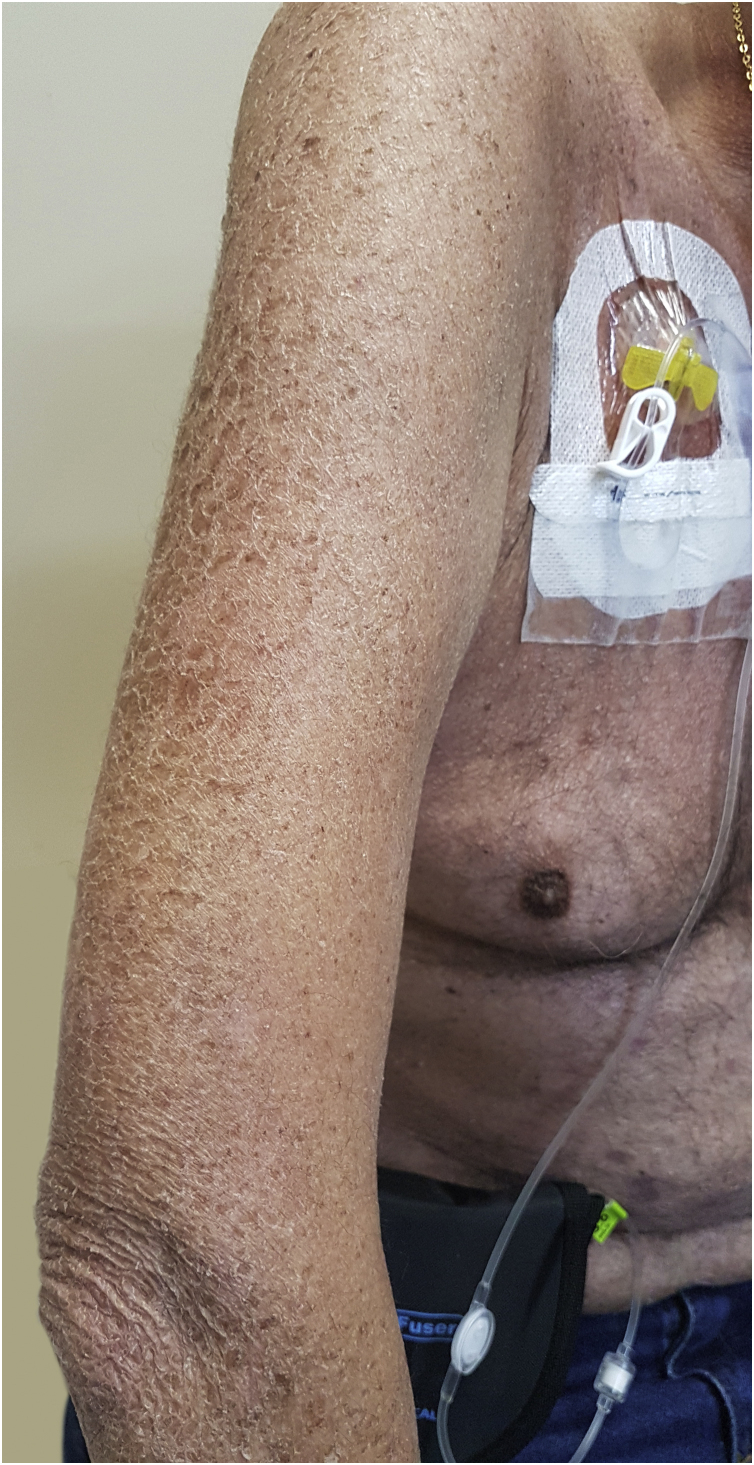
A 63-year-old patient using cetuximab for the treatment of colorectal carcinoma with severe xerosis.

The physiopathology of xerosis associated with targeted therapies, especially EGFR inhibitors, seems to be related to the process of epidermal differentiation and homeostasis. These drugs lead to increased inflammation, keratinocyte apoptosis, sensitivity to ultraviolet radiation, and altered differentiation, which would facilitate skin dryness (Fig. 4)^47^. This seems to progress towards the extremities, worsening with the time of treatment, the peak occurring around one to three months after starting the medication. Due to increased cutaneous fragility, pruritus, fissures formation, and local pain may occur, which can facilitate secondary infections^7^. Articles have concluded that the incidence of xerosis was higher with some drug classes, among them EGFR inhibitors^47^. Other authors report that cutaneous xerosis is present in up to 35% of the patients receiving EGFRi therapy^7^. Valentine et al. agree that EGFRi showed the highest rates of xerosis, but particularly mention panitumumab, with a total incidence of 47% of xerosis of all grades^47^.

### Paronychia

**Figure 5:**
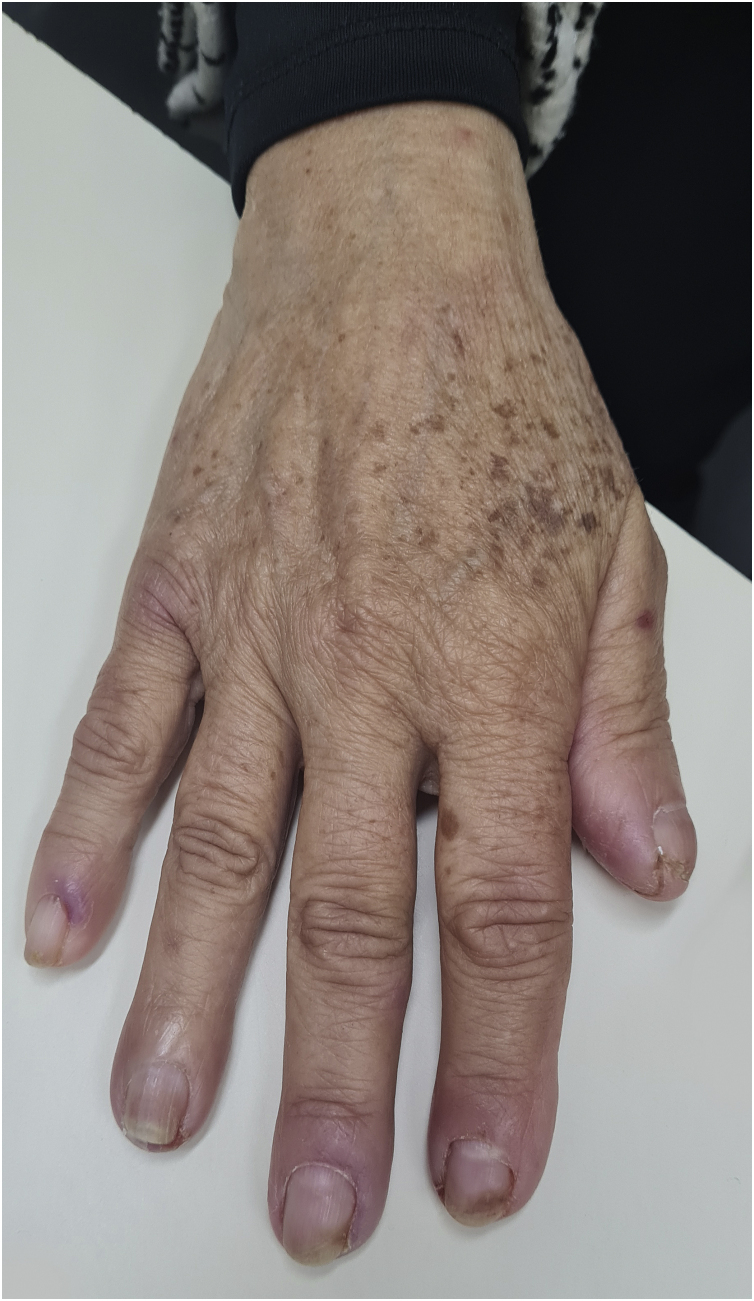
A 69-year-old patient using cetuximab with periungual changes which appeared two weeks after starting the medication.

**Figure 6:**
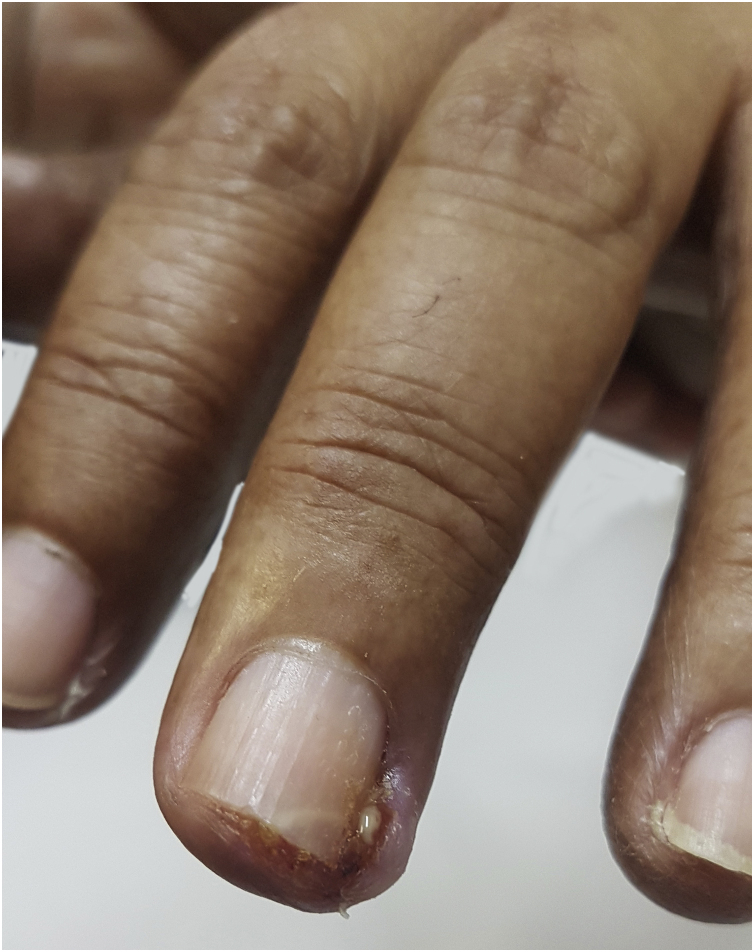
Periungual granuloma in a 56-year-old patient using cetuximab.

The physiopathological mechanism that leads to the development of these periungual lesions remains unknown. It is believed that the lesions result from the inhibition of EGFR and regulatory pathways of suprabasal keratinocyte proliferation, which would lead to changes in epidermal cell differentiation and migration associated with the inhibition of keratinocyte proliferation and decreased cell survival through apoptosis induction. As a result, the periungual epidermis becomes thinner and may be more susceptible to trauma, thus causing a similar reaction to inflammation secondary to the presence of a foreign body. The possibility of the participation of intracellular retinoid metabolism is also studied, as the use of isotretinoin and oral acitretin leads to the development of similar lesions^48^. It occurs approximately four to eight weeks after starting treatment but may appear up to six months later (Fig. 5). The literature describes the symptom onset between the 4^th^ and 8^th^ weeks after starting treatment with target therapies. Therefore, one can consider the onset of toxic effects to be a late event when compared to the others mentioned before^5^. It usually affects several fingers and formation of granulomas and periungual abscesses may occur. The first signs comprise a painful erythematous inflammation with edema and increased tenderness on the lateral nail folds. Bleeding and discharge from the site may also occur. As the condition progresses, friable granulation tissue may develop on the lateral nail folds, such as periungual granulomas. It can affect any finger or toe, but the thumbs and especially the halluces are the most often affected, probably because they are more susceptible to repeated microtrauma^5^. Pyogenic granuloma is a benign vascular tumor and it is considered a consequence of paronychia. Its high frequency in patients using these medications can be explained by repeated trauma and/or inflammation of the periungual tissues and the high vascularity of the nail unit (Fig. 6). Studies have suggested that there is a large number of vascular endothelial growth factor (VEGF) receptors at this location, which would allow more of these reactions to occur at these sites^49^. Paronychia and periungual granulomas are mostly reported in response to EGFRi, with an incidence close to 17%, including all grades of toxicity^5^, ^48^, ^49^.

### Hair changes

In mice, dysregulation of the EGFR-Ras-Raf pathway can result in abnormal hair follicle morphogenesis. However, it is not known why EGFR inhibitors paradoxically induce alopecia of the scalp but result in hirsutism and trichomegaly of eyebrows and eyelashes^50^. Hair changes usually manifest after two months of treatment. Scalp alopecia is typically inflammatory and may be cicatricial or non-cicatricial. However, spontaneous improvement of hair growth is observed after several months of continuous EGFRi therapy. Mild diffuse alopecia is relatively frequent with EGFR inhibitors (e.g., erlotinib, afatinib, cetuximab, panitumumab). As for cicatricial alopecia, it has been reported in 5% of patients treated with cetuximab, which is mentioned in the literature as a possible consequence of secondary bacterial infection of the scalp^50^. Paradoxically to alopecia in the scalp and extremities, facial hirsutism may occur^17^, with trichomegaly being a recent finding which occurs in up to 30% of patients using these medications^5^. It is mostly seen on eyelashes and presents within ten weeks of starting treatment^50^, ^51^. In a recent publication, Sartori DS et al. demonstrated with scanning electron microscopy that panitumumab-induced hair changes are pili canaliculi (longitudinal grooves)^52^.

### Changes in mucous membranes

Oral complications reported in patients treated with EGFRI are infrequent. The most commonly reported oral complication is mucositis^19^. Additionally, conjunctivitis has been reported in 6% to 20% of the patients^5^. The genitalia is occasionally affected, with vulvovaginitis sicca (especially in postmenopausal women) or balanitis^14^. More broadly, according to Lacouture, approximately 15% of those using EGFR TKI inhibitors develop stomatitis^5^.

## Conclusion

EGFRi constitute an important therapeutic modality in the treatment of cancer patients; however, they present cutaneous toxicity as one of their limitations, with acneiform eruptions being the most prevalent. The literature review on the mechanisms that may be related to the development of acneiform eruptions in patients using EGFRi, despite the need for further studies on the topic, suggests some associations. It is important for dermatologists to be aware of some risk factors for the development of lesions and thus provide early treatment for the patient and prevent cancer treatment abandonment, with a consequent worse prognosis.

## Financial support

None declared.

## Authors' contributions

Julia Kanaan Recuero: Approval of the final version of the manuscript; design and planning of the study; drafting and editing of the manuscript; collection, analysis, and interpretation of data; effective participation in research orientation; critical review of the literature; critical review of the manuscript.

Joana Roberta Fitz: Drafting and editing of the manuscript; collection, analysis, and interpretation of data; critical review of the manuscript.

Andrea Abe Pereira: Drafting and editing of the manuscript; collection, analysis, and interpretation of data; critical review of the manuscript.

Renan Rangel Bonamigo: Approval of the final version of the manuscript; design and planning of the study; drafting and editing of the manuscript; collection, analysis, and interpretation of data; effective participation in research orientation; critical review of the literature; critical review of the manuscript.

## Conflicts of interest

None declared.
