# Supplementary material for: EGFR inhibitors: clinical aspects, risk factors and biomarkers for acneiform eruptions and other mucosal and cutaneous adverse effects
Source: An Bras Dermatol. 2023 Mar 27;98(4):429–39. doi: 10.1016/j.abd.2022.10.004 (PMC10334360; doi:10.1016/j.abd.2022.10.004)
Supplement: Supplementary file 1 [file mmc1.docx]

Appendix

| **Questions** |
| --- |
| 1. Epidermal growth factor receptors are found in the following structures of the cutaneous integument: |
| a) Keratinocytes, exclusively |
| b) Glandular skin appendages, exclusively |
| c) Capillary endothelium, exclusively |
| d) In all the abovementioned structures |
|  |
| 2. Regarding epidermal growth factor receptor inhibitors (EGFRi), which sentence is wrong: |
| a) They are not used in lung neoplasms |
| b) They are used in colorectal and breast cancer |
| c) They can be classified into monoclonal antibodies and inhibitors: tyrosine kinase, EGRF and HER2, erB receptors, multikinases, VEGFR and RET oncogene |
| d) The inhibitor cetuximab is a monoclonal antibody against EGFR |
|  |
| 3. The most common skin toxicity caused by EGFRi is: |
| a) Alopecia |
| b) Paronychia |
| c) Papulopustular (acneiform) eruption |
| d) Xerosis |
|  |
| 4. Regarding papulo-pustular eruptions caused by EGFRi: |
| a) They usually start within the first week of use |
| b) Follicular infiltration is always lymphocytic |
| c) There is p53 expression in the basal keratinocytes and hair follicles and the lesions initially do not show microbial overpopulation. |
| d) This adverse effect never causes the discontinuation of treatment, as it is considered of mild severity |
|  |
| 5. Regarding the risk factors for the development of papulo-pustular eruptions related to the use of EGFRi: |
| a) The use of higher doses is not important as a risk factor |
| b) Monoclonal antibodies constitute a more frequent cause than tyrosine kinase inhibitors |
| c) Elderly and female patients are more likely to be affected |
| d) Biomarkers have been ruled out as useful in predicting acneiform eruptions |
|  |
| 6. In the management of acneiform reactions induced by EGFRi: |
| a) It can be performed prophylactically with systemic steroids |
| b) The use of topical steroids should always be avoided |
| c) Doxycycline is an antibiotic to be used in doses of up to 200 mg/day |
| d) The withdrawal of the EGFRi is the only measure to be taken |
|  |
| 7. In the evaluation and management of papulopustular eruptions while using EGFRi: |
| a) Those with a bacterial etiology present a rapid onset, after up to one week. |
| b) Topical should be the drug of choice in the later stages of the eruption |
| c) The follicular eruption should not be considered of bacterial etiology when appearing after the fourth week of evolution |
| d) Sterile pustules are more common in the early stages of the eruption |
|  |
| 8. In the Common Terminology Criteria for Adverse Events (CTCAD) classification: |
| a) There are four grades of acneiform eruption severity |
| b) The existence of papules and/or pustules covering > 30% of body surface defines Grade 4 severity |
| c) The classification involves morphological aspects and extension, and does not cover symptoms and psychosocial impact |
| d) There are five grades of severity |
|  |
| 9. The assessment of serum creatine kinase (CK) in EGFRi users: |
| a) Was shown to be unimportant |
| b) Demonstrates that there is an association between its increase and eruption severity |
| c) If reduced, the use of the medications should be contraindicated |
| d) They does not seem to have a predictive role for the development of skin lesions |
|  |
| 10. Regarding hair alterations caused by EGFRi: |
| a) Alopecia or hypertrichosis may occur |
| b) Cicatricial alopecia occurs in most patients |
| c) It usually occurs in plaques like alopecia areata |
| d) The onset period is short, approximately two weeks. |
|  |
| Correct answers: 1d, 2a, 3c, 4c, 5b, 6c, 7d, 8d, 9b, 10a |
